# Lack of atrial branch perfusion during acute ischemia is not associated with new-onset atrial fibrillation during STEMI

**DOI:** 10.1016/j.ijcha.2025.101668

**Published:** 2025-04-26

**Authors:** Marina M. Demidova, Goran Olivecrona, Anton P.M. Gorgels, David Erlinge, Pyotr G. Platonov

**Affiliations:** a Department of Cardiology, Clinical Sciences, Lund University, Lund, Sweden; b Department of Cardiology, Maastricht University, Maastricht, the Netherlands

**Keywords:** STEMI, Atrial infarction, Atrial fibrillation

## Abstract

**Background and aim:**

Atrial fibrillation (AF) often complicates ST-elevation myocardial infarction (STEMI). Atrial ischemia due to non-perfused atrial branches may contribute to its underlying mechanisms. We aimed to assess the association between atrial branches perfusion during STEMI and AF occurrence during and after STEMI.

**Methods:**

We performed a single-center retrospective register-based cohort study. Consecutive STEMI patients admitted for percutaneous coronary interventions (PCI) during 2007–2010 were included (n = 1960, age 65 ± 12 years, 71 % male) and followed up for 10 years. Clinical characteristics were retrieved from the Swedish national registries. ECGs recorded before, during or after STEMI were exported from a digital archive. Patients with AF documented prior to STEMI and AF after CABG during hospitalization for STEMI were excluded. The endpoint was the first AF episode either during hospitalization or after discharge.

**Results:**

Non-perfused atrial branches were observed in 59 out of 212 proximal RCA occlusions and in 4 out of 93 proximal LCX occlusions. All other culprit vessels (n = 1,655) were presumed to be unrelated to atrial perfusion. The absence of atrial branch perfusion was not associated with new-onset AF either during hospitalization or after discharge (HR = 0.79, 95 % CI 0.35–1.78, p = 0.570).

**Conclusion:**

The lack of atrial branch perfusion during STEMI was not associated with new-onset AF either during or after STEMI.

## 1 Introduction

Atrial fibrillation (AF) complicates the course of ST-elevation myocardial infarction (STEMI) in 5–14 % of patients. [1], [2] Factors predisposing to AF in STEMI may be complex and can include acute ischemia, hypokalemia, increased vagal tone, endogenous or exogenous catecholamines, left ventricular dysfunction and inflammation superimposing on the underlying atrial substrate. [3] Increased atrial pressure leading to atrial dilation, as well as atrial volume overload, can contribute to possible underlying mechanisms of AF. [4] In addition, atrial ischemia and infarction may occur alongside ventricular infarction, leading to significant atrial dilation and subsequent fibrosis, which increases the risk of atrial arrhythmias. Few clinical case studies with autopsy verification of atrial infarction reported a high rate of atrial arrhythmias;[5], [6] however, the association between atrial ischemia and AF in unselected cohorts of STEMI patients with angiographic verification of impaired supply to atria has, to the best of our knowledge, not been studied.

Our aim was to assess the association between atrial branches perfusion during STEMI and AF occurrence during and after STEMI.

## 2 Methods

### 2.1 Study population and data sources

We performed a single-center retrospective register-based cohort study. Consecutive STEMI patients admitted for primary percutaneous coronary interventions (PCI) to Lund University Hospital, Sweden during 2007–2010 were included.

The Swedish Web System for Enhancement and Development of Evidence-based care in Heart disease Evaluated According to Recommended Therapies (SWEDEHEART) registry was used to assess demographic and clinical characteristics. Information regarding the first date of AF diagnosis (ICD-10 code I48) was obtained from the Swedish National Patient Register (SNPR). Mortality outcomes was obtained from the Swedish National Patient Register (SNPR), and the Swedish Cause of Death Registry (SCDR).

Skåne Region digital ECG archive, which contains all ECGs recorded in the Skåne Region during hospital admissions and in primary care facilities from 1988 onward, was used as the source for ECG-verification. For every included patient, all available ECGs recorded at any time before, during or after STEMI (n = 58,907) and stored in digital format were retrieved from either the GE Marquette MUSE system (GE Medical Systems, Milwaukee, Wisconsin) or the Infinity MegaCare ECG Management System (Dräger, Lübeck, Germany) databases. ECGs were then automatically analyzed using the Glasgow algorithm. [7], [8], [9] Then we performed an ECG search by first preselecting diagnostic codes generated by the automatic algorithm that utilizes regularity logic. These codes may be assigned to an AF ECG, such as “atrial fibrillation”, “probable atrial fibrillation”, “possible atrial flutter”, “possible ectopic atrial rhythm” and “undetermined rhythm”. For each patient, all preselected ECGs were manually reviewed and only verified AF ECGs were used for further analysis.

**Fig. 1:**
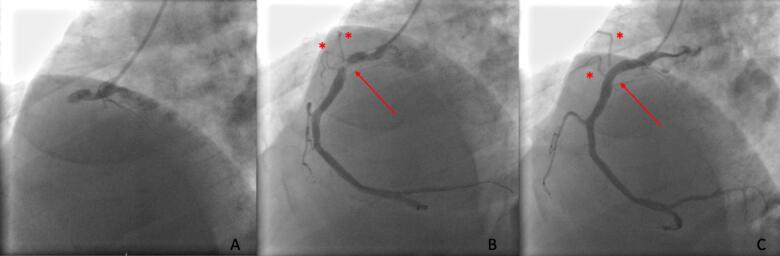
Coronary angiography images before (A), during (B) and after (C) primary PCI of RCA. A-proximal RCA occlusion; B- two atrial branches are revealed to arise from previously occluded segment of RCA (arrow); C-normalized flow in RCA and atrial branches (asterixis) after stent deployment.

AF prior to STEMI was documented by record linkage with the Swedish National Patient Register and review of ECGs recorded before STEMI from the regional digital ECG archive; patients with pre-existing AF (paroxysmal, persistent or permanent) were excluded (Fig. 1). Due to the specific underlying conditions and the high occurrence rate of AF after CABG, patients with AF first documented during or after an acute coronary artery bypass grafting (CABG) procedure during the index hospitalization were also excluded from the analysis.

Angiographic data were obtained from the SWEDEHEART register. In patients with proximal right coronary artery (RCA) occlusion (1st segment) or left circumflex artery (LCx) occlusion (11st segment), angiograms stored in hospital digital database were reviewed to assess perfusion of atrial branches before and after PCI. In total, 305 angiograms were independently evaluated by two operators, with a consensus decision used for the final classification of the presence and perfusion of the atrial branch. All other culprit vessels were presumed to be unrelated to perfusion of atrial branches. The patients in whom the perfusion of atrial branches could not be validated, were excluded from the analysis.

### 2.2 Study endpoints

The primary endpoint was the first AF episode after STEMI symptom onset.

The primary endpoint was identified through the crosscheck of: (1) the SWEDEHEART Registry and SNPR, (2) the ECG digital database, and (3) medical histories, including heart rhythm surveillance telemetry records. AF was assessed for the period starting from STEMI symptom onset ending December 31st 2018.

The secondary endpoints were total mortality, cardiovascular mortality, and combined endpoint including AF or death from any cause.

### 2.3 Statistical analysis

The association between non-perfused atrial branches and primary and secondary endpoints was tested in the Cox-regression analysis adjusted for demographic covariates. The Kaplan-Meier method was used to generate survival curves indicating freedom from atrial fibrillation during follow-up.

Continuous variables are presented as mean ± standard deviation or median and 25 %-75 % as appropriate depending on distribution. Variables were compared across groups using chi-square test for categorical variables and Student’s *t*-test for continuous variables with an approximate normal distribution, or Mann-Whitney *U* test, as appropriate, without imputation of missing data. P values < 0.05 were considered significant. SPSS 28.0 package (SPSS Inc., Chicago, Illinois, USA) was used for analysis.

The study was approved by the Regional Ethics Committee in Lund, Sweden (# 2010/585).

## 3 Results

During the period from 2007 to 2010, 2276 consecutive STEMI patients were admitted for primary PCI. After excluding patients in whom atrial branch perfusion could not be verified (n = 134), patients with pre-existing AF prior to STEMI (n = 177), and patients who developed AF during or after acute CABG within index hospitalization for STEMI (n = 5), 1,960 patients remained eligible for final analysis; their clinical characteristics were shown in Table1. Patients with non-perfused atrial branch were older and more often have hypertension compared to patients with normally perfused atrial branch.

**Table 1:** Clinical characteristics.

Characteristic	All patients (n = 1,960)	Perfused atrial branches (n = 1,897)	Non-perfused atrial branches (n = 63)	p-value
Age, years	65 ± 12	65 ± 12	69 ± 10	0.002
Male gender, %	1,387 (70.8 %)	1,348 (71.1 %)	39 (61.9 %)	0.122
BMI	27.0 ± 4.5	27.0 ± 4.6	25.9 ± 3.7	0.056
Medical history:				
Hypertension	745 (38.2 %)	711 (37.6 %)	34 (55.7 %)	0.005
Diabetes	235 (12.0 %)	226 (11.9 %)	9 (14.8 %)	0.546
MI history	258 (13.2 %)	249 (13.2 %)	9 (14.5 %)	0.704
PCI history	189 (9.7 %)	185 (9.8 %)	4 (6.5 %)	0.513
CABG history	65 (3.3 %)	64 (3.4 %)	1 (1.6 %)	0.721
Chronic HF	44 (2.2 %)	42 (2.2 %)	2 (3.2 %)	0.651
Stroke	110 (5.6 %)	106 (5.6 %)	4 (6.5 %)	0.776
Smoking	686 (36.5 %)	659 (36.2 %)	27 (45.0 %)	0.174
Multivessel disease	1,019 (55.3 %)	978 (54.9 %)	41 (67.2 %)	0.066
Inferior MI	1,052 (55.0 %)	989 (53.5 %)	32 (100 %)	<0.001
Symptom-PCI time, min	213 [141–385]	213 [141–385]	202 [135–295]	0.685
LV EF < 30 %	134 (7.0 %)	132 (7.0 %)	2 (3.2 %)	0.314
Killip II, III, IV	136 (7.0 %)	131 (6.9 %)	5 (7.9 %)	0.799

BMI – body mass index; MI – myocardial infarction; PCI – percutaneous coronary intervention; CABG-coronary artery bypass grafting; HF – heart failure; LV EF – left ventricle ejection fraction.

**Fig. 2:**
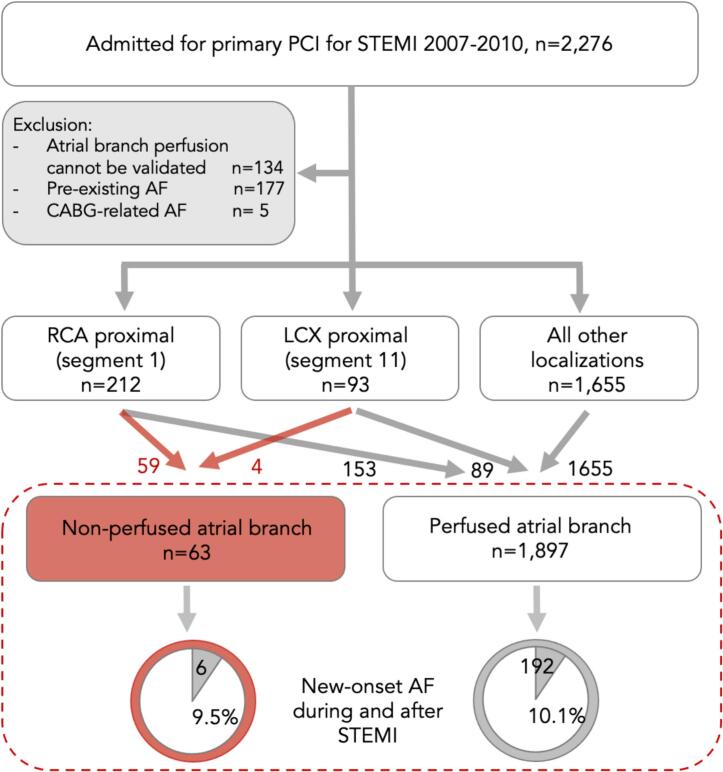
Patient flow chart. AF – atrial fibrillation; CABG – coronary artery bypass grafting; LCX – left circumflex coronary artery; PCI – percutaneous coronary intervention RCA – right coronary artery; STEMI – ST-elevation myocardial infarction.

Left main coronary artery was the infarct-related artery in 2 (1.1 %) patients, and the left anterior descending artery and diagonal branches – in 767 (39.1 %) patients. The culprit LCX and branches were observed in 241 (12.3 %) patients, with proximal LCX lesion in 93 cases (4.7 %). RCA and its branches were the culprits in 719 (36.7 %) patients, and proximal RCA − in 212 (10.8 %) patients. Non-perfused atrial branches were observed in 59 out of 212 proximal RCA occlusions and in 4 out of 93 proximal LCX occlusions (Fig. 1, Fig. 2). In 48 % of patients, atrial coronary branches originated from RCA, in 5 % − from LCX, and multiple coronary atrial branches both from RCA and LCX were identified in 47 % of patients. In all 63 cases of non-perfused atrial branches, perfusion was successfully restored following PCI.

**Fig. 3:**
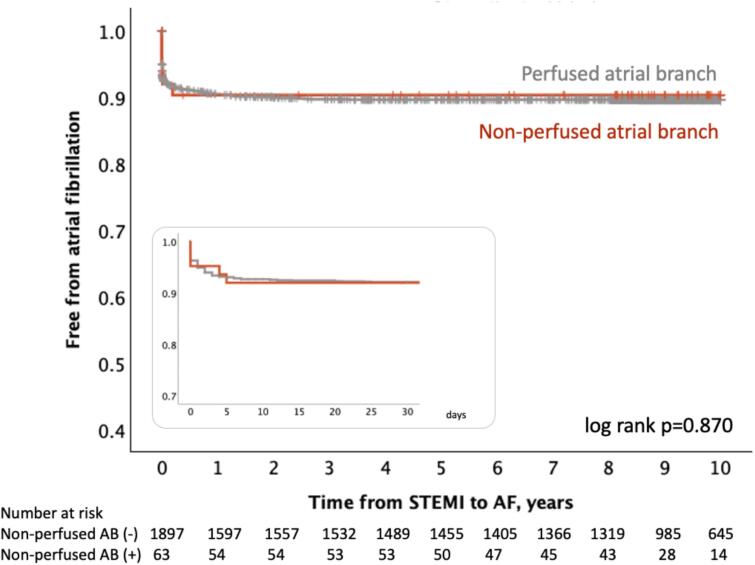
Kaplan-Meier survival curves indicating freedom from AF in STEMI patients with regard to the presence of non-perfused atrial coronary branch in acute period of STEMI (patients with pre-existing AF were excluded, patients in whom the perfusion of atrial branches could not be validated were also excluded), (n = 1,960). Insert shows freedom from AF during the first 30 days of follow-up. AB – atrial branch; AF – atrial fibrillation;

Out of 1,960 patients without AF prior to STEMI, AF occurred between STEMI symptom onset and hospital discharge in 144 patients, within 30 days in 152 patients, and by the end of follow-up in 198 patients. Median follow-up time for AF was 3325 [2062–3650] days. Lack of atrial branch perfusion was not associated with new-onset AF during hospitalization (OR = 0.92, 95 % CI 0.36–2.40, p = 0.871), within the first 30 days (HR = 0.87, 95 % CI 0.36–2.13, p = 0.761), or during the 10-year follow-up period (HR = 0.79, 95 % CI 0.35–1.78, p = 0.570) (Fig. 3).

Median length of follow-up for total and cardiovascular death was 3393 [2931–3650] days. The absence of atrial branch perfusion was not associated with total mortality (HR = 1.11 95 %CI 0.74–1.67, p = 0.624), cardiovascular mortality (HR = 1.26 95 % CI 0.75–2.12, p = 0.385) or reaching the combined endpoint including AF or death from any cause (HR = 1.01 95 %CI 0.68–1.49, p = 0.980) either.

## 4 Discussion

In our study of a large unselected population of STEMI patients with angiographic verification of coronary atrial branch perfusion, we found no evidence that non-perfused atrial branches are associated with either new-onset AF during STEMI or AF during follow up.

Most studies on the association between atrial infarctions and atrial arrhythmias are case reports that describe ECG findings [10] with autopsy confirmation. [5], [6] There is very limited angiographic data on atrial coronary circulation in patients with myocardial infarction. For instance, a study based on the APEX-AMI population of STEMI patients used ECG criteria for diagnosing atrial infarction, but did not report angiographic findings related to atrial blood supply. [11] Angiographic descriptions of atrial infarction are mostly limited to case reports and series.[12] In one study on patients undergoing elective PCI, a higher occurrence of AF and other atrial arrhythmias was associated with experienced accidental occlusion of an atrial branch during stent implantation in close proximity of coronary atrial branch origin.[13] In available literature, we found only one study that systematically described angiographic findings regarding atrial branches in patients with acute coronary syndrome,[14] and our view on complexity of atrial coronary circulation is similar with that study.

The main reason for the absence of perfusion in the coronary atrial branch was acute occlusion of the coronary artery proximal to the origin of the main atrial branch. Non-perfusion of the coronary atrial branch was most frequently observed in cases of proximal RCA occlusion. The main atrial branch originates less frequently from the LCX than from the RCA. In cases of LCX occlusion, the site of occlusion was more distal to the atrial branch origin in most cases, allowing the atrial branch to remain patent. In some cases, atrial branches originating from both the RCA and LCX were observed, and occasionally, small multiple atrial arteries arising from the RCA were found. These anatomical variations in atrial coronary circulation, with common multiple vessel supply, may partly explain why the clinical presentation, electrocardiographic (ECG) abnormalities, and arrhythmic complications of a non-perfused atrial branch are unpredictable and inconsistent. Additionally, oxygen diffusion from highly oxygenated blood in the left atrial cavity into the thin atrial wall may limit the extent of left atrial infarctions.[15], [16].

While planning the study, we hypothesized that atrial infarction could result in severe fibrotic replacement within the atria and impaired intra-atrial conduction, potentially contributing to the development of AF during follow up after discharge. Experimental studies have demonstrated that atrial infarction may indeed be associated with an increased susceptibility to AF, as it promotes atrial spontaneous ectopy and creates conduction abnormalities that sustain reentry. [17], [18] However, it is important to consider that in the aforementioned experimental studies, isolated atrial myocardial infarction was induced, whereas in clinical settings of STEMI, ventricular myocardial infarction may be the predominant underlying pathology, potentially diminishing the contribution of atrial ischemia to the risk of new-onset AF. On the other hand, it is possible that the formation of electrically inactive tissue in the atrial wall has a protective effect against atrial arrhythmias following STEMI.

### 4.1 Limitations

Some AF episodes during follow-up that occurred outside the Skåne region and were not covered by the regional ECG database may be underreported. Silent AF during follow-up may also be underreported, as ambulatory ECG monitoring was not routinely performed after discharge.

Echocardiographic data were not routinely collected for this STEMI patient cohort. Consequently, data on left atrial size, which has been previously shown to predict AF in various clinical settings, were not available.

## 5 Conclusion

In a large non-selected population of STEMI patients admitted for primary PCI, the lack of atrial branch perfusion was not associated with new-onset AF during or after STEMI.

Funding.

This study was supported by the Swedish Heart Lung Foundation (#20180222 to M.M.D. and #20200674 to P.G.P.), Donation funds at the Skane University Hospital (grant #96336, P.G.P.), Governmental funding of clinical research by the Swedish National Health Service (ALF, grant #46702, P.G.P.) and Eva and Carl-Eric Larssons Foundation (MMD and PPG).

## CRediT authorship contribution statement

**Marina M. Demidova:** Writing – review & editing, Writing – original draft, Methodology, Investigation, Formal analysis, Data curation, Conceptualization. **Goran Olivecrona:** Writing – review & editing, Validation. **Anton P.M. Gorgels:** Writing – review & editing, Conceptualization. **David Erlinge:** Writing – review & editing, Resources. **Pyotr G. Platonov:** Writing – review & editing, Supervision, Project administration, Methodology, Funding acquisition, Conceptualization.

## Declaration of competing interest

The authors declare the following financial interests/personal relationships which may be considered as potential competing interests: [Marina M Demidova reports financial support was provided by Heart and Lung Foundation. Pyotr. Platonov reports financial support was provided by Heart and Lung Foundation. If there are other authors, they declare that they have no known competing financial interests or personal relationships that could have appeared to influence the work reported in this paper].
